# The Week's Work

**Published:** 1909-11-13

**Authors:** 


### The Week's Work.

### THE KING AND THE HOSPITALS.

Within the past week the King has repeatedly shown how closely he is interested in hospital work. On Thursday, November 4, he opened the extension of the National Hospital for the Paralysed and Epileptic (Albany Memorial), Queen Square, W.C., and conferred the honour of knighthood upon Mr. F. Macmillan, the chairman of the Board of Management. On arriving at the hospital buildings the King was received by their Royal Highnesses the Duchess of Albany and Princess Alexander of Teck (the president and vice-president of the Jubilee Fund) and the staff and friends of the institution, and was conducted to the daïs erected in the Out-patients' Hall, where the Reception Committee had assembled, the principal governors of the hospital, contributors to and helpers of the Jubilee Fund, and members of the Board of Management and Jubilee Committee; the consulting staff and distinguished visitors being assembled in the body of the hall. The National Anthem was sung, and after the Bishop of London had offered up prayer the Chairman of the Board of Management read an address to which His Majesty was graciously pleased to reply, and declared the new building open, the band in the Out-patients' Hall playing "God Save the King." Afterwards the King was conducted through the new buildings, and also through a ward on the ground floor, before returning to the Palace. We congratulate H.R.H. the Duchess of Albany and the authorities upon the results of the Jubilee appeal, and the arrangements made to secure the success of the Jubilee meeting, the King's approval of which was expressed by the knighthood conferred upon Sir Frederick Macmillan. Mr. Hamilton, the secretary, and his staff, are also entitled to recognition and warm thanks, for they must have put a great deal of extra work and energy into the organisation, which has yielded sufficient money to defray the cost of the Jubilee extensions (£10,000), and to add £15,000 to the reserve fund. On the following Saturday afternoon His Majesty and the Queen carried out their promise to visit the Art Loan Exhibition which is being held at Lynn in aid of the funds of the West Norfolk and Lynn Hospital. Their Majesties were accompanied by the Queen of Norway and Princess Victoria, and, in accordance with the King's wish, there were no official ceremonies, the visit being considered a private one.

### WHAT OF THE FOUNDERS?

The proceedings at the opening ceremony of the National Hospital for the Paralysed have created a good deal of interest and some criticism. It is remarkable, for instance, that the names of the distinguished guests did not include those of the men who made this hospital largely what it is to-day, and who were associated with it and worked hard for it for decades of years. Is it possible that by some oversight invitations were not sent to the founders or their representatives, although all of them were governors, and some of them, at any rate, are alive to-day? We do not find any mention, for instance, of the Chandler family, at whose instance this hospital was originally founded at the Mansion House, of General Porter, who was, we believe, the most active cause of the rebuilding of this hospital on its present site, or of Mr. B. Burford Rawlings, who for nearly thirty-five years, as recorded in The Hospital of July 20, 1902, devoted the best of his energies and the whole of his time to the work of building up the greatest of English hospitals for the paralysed. There are others who might be mentioned, but the names given suffice to show that there is ground for the surprise expressed that the men, or some of them, to whose work this hospital largely owes its existence, should not have been present in person or by their representatives at its Jubilee, which His Majesty the King attended in person. There can be no question that of all institutions this hospital is one where the Jubilee celebrations should have been made the occasion to unite everyone interested in its work, and so to consolidate its prosperity and strength. We are quite sure that H.R.H. the Duchess of Albany, as President of the Jubilee Committee, would have been the first to wish that an invitation to attend on this memorable occasion should have been extended to every person living who has had a hand in building up this noble hospital.

### RESEARCH AND RESPONSIBILITY.

There can be no question, as we have shown, that in these days the increase in the number of patients afflicted by paralysis and nervous diseases renders it essential that much more accommodation shall be provided for such cases than at present exists in this country. Possibly one reason for the relative smallness of the number of hospital beds for nervous diseases to-day is the feeling, which was much more prevalent when the National Hospital for Paralysis was founded, that patients suffering from idiocy and other similar maladies, are not eligible for treatment in a hospital. Research work has, however, extended so enormously, especially in recent years, that the history of this hospital records, we believe, an instance where a distinguished surgeon sent in such a case for admission and objection was raised by a reference to the rules. This incident, unless we were misinformed, was the origin of the friction which led to the inquiry and the adoption of new regulations. Assuming such to be the case we have here a hospital where the responsibility of silence is more than usually grave, and this remark must apply not only to the medical staff of all grades but to each member of the general committee. The King in his reply to the address wisely insisted that, "Sufferers from paralysis and epilepsy are so helpless, so utterly dependent for their very existence on the exertions of others, that without such institutions as the National Hospital many of the poor . . . . must inevitably be reduced with their families to the greatest misery." His Majesty expressed his conviction that its great work in future will be carried on even more efficiently than in bygone years, a conviction which we have no doubt the members of the medical staff and of the committee of management will make it their business to justify by results.

### SCHOOL CHILDREN AND HOSPITAL TREATMENT.

The results of the negotiations between the Education Committee of the London County Council and the London hospitals with reference to the medical treatment of children have been issued. The Sub-Committee of the Day Schools has proposed that an experiment for twelve months be undertaken by which, at a cost of £4,000 a year, 16,000 children will have the benefit of medical treatment in respect of diseases of the eye, ear, and skin at the following eight hospitals: The Belgrave Hospital for Children (for not less than 1,760 children); Charing Cross Hospital (for not less than 2,200 children); Hospital for Diseases of the Throat, Golden Square (for not less than 1,000 children); King's College Hospital (for not less than 500 children); London Hospital (for not less than 4,750 children); Metropolitan Ear, Nose, and Throat Hospital (for not less than 500 children); Royal London Ophthalmic (Moorfields) Hospital (for not less than 3,000 or more than 6,000 children); St. George's Hospital (for not less than 2,000 children). It is further proposed to accept the offer of the committee of the Charing Cross Hospital to provide treatment by means of *x*-rays for 25 children attending public elementary schools at a cost not exceeding £35. These proposals will be subject to the approval of the Board of Education, and still leave 5,000 children unprovided for. The cost of a complete scheme for the treatment of the three diseases mentioned is expected to cost from £5,000 to £6,000 per annum. It is hardly likely, however, that the scheme will take practical shape before January 1; the cost till then is not estimated to be more than £1,000.

### THE MIDHURST SANATORIUM.

Our contemporary, *John Bull*, which has already drawn attention to the rather unnecessarily strict rules prevailing at a well-known consumption sanatorium, now attempts to work up a case against the King Edward VII. Sanatorium at Midhurst. At any rate, it gives full publicity to a letter from " An Ex-Patient," in which certain specific charges are made against the management of the institution, and on the strength of these it demands an immediate inquiry into the indignities " resulting from the system of petty officialdom " which patients have to bear with. The first part of the indictment deals with the routine, which, enumerated in detail in the unsympathetic and somewhat ungrammatical fashion of an " Ex-Patient," sounds uninviting enough to the lay reader. But we may point out that even in this indictment there is nothing to which serious objection can be taken. Patients who come to a consumption sanatorium must be prepared to undergo these " hardships " and " indignities." They must be prepared to find the medical attendant relying much on their temperatures in outlining their day's work, and they must bear with such indignities, if indignities they be, of getting up and going to bed at stated hours. They must even bear with " unholy draughts," however much they may like to live in the ordinary atmosphere of the average dwelling-house. Nor can any patient be a judge of the fitness or unfitness of his fellow-patients for treatment. The allegations that certain " cured " patients are kept, occupying rooms and beds which should be available for others waiting admission, may therefore be entirely dismissed. Increase of body weight can hardly be accepted as sufficient proof of cure to warrant the assertion that a man who " put on two stones weight in eight weeks " was fit to be discharged.

### MORE SERIOUS CHARGES.

While the main part of the indictment drawn up by " An Ex-Patient " is frivolous and unimportant, there are some points in it which demand attention. If it is true, for instance, that " the writing-room may only be used after 4.30 p.m., the post leaving at 5.45 p.m., so a room that accommodates but five people is only available for an hour a day for sixty men to write their letters—and writing elsewhere is strictly forbidden "—the management should see to it that this obnoxious and absurd rule is at once altered. The more serious allegation that patients suffering from " a kind of ringworm," or from scabies, are allowed to mingle with the others, and that this laxity has occasioned an epidemic of the diseases, demands an explanation from the authorities, to say the least. A further serious charge is that the rooms are damp and wet, that the radiators and heating system are inefficient, and that patients, in consequence, have to endure a great deal of discomfort and annoyance. " The situation of the building," we are informed, " is exceedingly damp and misty . . . This mist saturates everything in and about the place; even one's pillows frequently need airing before turning in, then only to be saturated again during the night; and the only way to ensure anything like dry underclothing, or night attire, is to keep a hot-water bottle in a drawer or wardrobe continuously." These points require some attention from the authorities, for allegations like these, however unsupported, do an incalculable amount of harm if not contradicted. Doubtless many former patients can be found who will contradict these statements made by the " Ex-Patient ": during the past few days we have discussed the subject with two such patients, who, beyond confirming the assertion that there is a certain amount of rigour in enforcing the routine of the institution, have nothing to say in support of these graver allegations to which we have referred. Our contemporary is perfectly justified, as a lay paper without means of testing the statements, in publishing the letter. To demand an inquiry, and take the counts as proven, however, before the authorities have had a chance of replying to the criticism, cannot be either justifiable or fair to the institution, which, from all accounts, is doing excellent work in an excellent manner.

